# Optimal Down Regulation of mRNA Translation

**DOI:** 10.1038/srep41243

**Published:** 2017-01-25

**Authors:** Yoram Zarai, Michael Margaliot, Tamir Tuller

**Affiliations:** 1 School of Electrical Engineering, Tel-Aviv University, Tel-Aviv 69978, Israel; 2 Sagol School of Neuroscience, Tel-Aviv University, Tel-Aviv 69978, Israel; 3 Dept. of Biomedical Engineering, Tel-Aviv University, Tel-Aviv 69978, Israel

## Abstract

Down regulation of mRNA translation is an important problem in various bio-medical domains ranging from developing effective medicines for tumors and for viral diseases to developing attenuated virus strains that can be used for vaccination. Here, we study the problem of down regulation of mRNA translation using a mathematical model called the ribosome flow model (RFM). In the RFM, the mRNA molecule is modeled as a chain of *n* sites. The flow of ribosomes between consecutive sites is regulated by *n* + 1 transition rates. Given a set of feasible transition rates, that models the outcome of all possible mutations, we consider the problem of maximally down regulating protein production by altering the rates within this set of feasible rates. Under certain conditions on the feasible set, we show that an optimal solution can be determined efficiently. We also rigorously analyze two special cases of the down regulation optimization problem. Our results suggest that one must focus on the position along the mRNA molecule where the transition rate has the strongest effect on the protein production rate. However, this rate is not necessarily the slowest transition rate along the mRNA molecule. We discuss some of the biological implications of these results.

Gene expression is the process by which the genetic code inscribed in the DNA is transformed into proteins. The process consists of four main steps: *transcription* of a DNA gene into an mRNA molecule, *translation* of the mRNA molecule to a protein, degradation of mRNA molecules, and degradation of proteins. During mRNA translation, macromolecules called ribosomes move unidirectionally along the mRNA molecule, decoding it codon by codon into a corresponding chain of amino acids that is folded to become a functional protein. Translation is a fundamental biological process, and understanding and re-engineering this process is important in many scientific disciplines including medicine, evolutionary biology, and synthetic biology^1^.

New methods that measure gene-specific translation activity at the whole-genome scale, like polysome profiling^2^ and ribosome profiling^3^, have led to a growing interest in mathematical models for translation. Such models can be used to integrate and explain the rapidly accumulating biological data as well as to predict the outcome of various manipulations of the genetic machinery. Recent methods that allow *real*-*time imaging* of translation on a single mRNA transcript *in vivo* (see, e.g. refs 4, 5, 6, 7) are expected to provide even more motivation for developing and analyzing powerful dynamical models of translation.

Down-regulation of translation is important in cell biology, medicine, and biotechnology. Indeed, diverse viruses down regulate host translation by cleaving proteins such as eIF4G^8^ in order to free more resources for translating the viral genes. As another example, in many organisms small RNA genes, such as microRNAs, hybridize to the mRNA in specific locations^9^,^10^ in order to down-regulate translation initiation or elongation^11^,^12^ and/or promote mRNA degradation. Alterations in the expression of microRNA genes contribute to the pathogenesis of most, if not all, human malignancies^13^, and many times cancer cells are targeted via generating tumor specific RNA interference (RNAi) genes that down-regulate the oncogenes^14^,^15^,^16^. Furthermore, many viral therapeutic treatments and viral vaccines are based on the attenuation of mRNA translation in the viral genes^17^,^18^,^19^,^20^,^21^. Down regulation of mRNA translation in an *optimal* manner is also related to fundamental biomedical topics such as molecular evolution and functional genomics^22^,^23^,^24^.

Here we study for the first time optimal down regulation of translation in a dynamical model of translation. A standard model for translation is the *totally asymmetric simple exclusion process* (TASEP)^25^,^26^. In this model, particles hop unidirectionaly along an ordered lattice of *N* sites. Simple exclusion means that a particle cannot hop into a site that is already occupied by another particle. This models hard exclusion between the particles, and creates an indirect coupling between the particles. Indeed, if a particle remains in the same site for a long time then all the particles preceding this site cannot move forward leading to a “traffic jam”. The hops along the lattice are stochastic, and the rate of hoping from site *i* to site *i* + 1 is denoted by *γ*_*i*_. A particle can hop to [from] the first [last] site of the lattice at a rate *α* [*β*]. The flow through the lattice converges to a steady-state value that depends on *N* and the vector of parameters


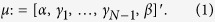


In the context of translation, the lattice is the mRNA molecule; the particles are the ribosomes; and hard exclusion means that a ribosome cannot move forward if the codon in front of it is covered by another ribosome. In the *homogeneous* TASEP (HTASEP) all the transition rates within the lattice are assumed to be equal and normalized to 1, i.e. *γ*_*i*_ = 1, 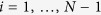, and thus the model is specified by an input rate *α*, an exit rate *β*, and an order *N*. TASEP is a fundamental model in non-equilibrium statistical mechanics that has been used to model numerous natural and artificial processes including traffic flow, surface growth, communication networks, evacuation dynamics and more^27^,^28^.

The *ribosome flow model* (RFM)^29^ is a nonlinear, continuous-time compartmental model for the unidirectional flow of “material” along a chain of *n* consecutive compartments (or sites). It can be derived via a mean-field approximation of TASEP^27^,^30^. In the RFM, the state variable 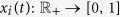, 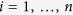, describes the normalized amount (or density) of “material” in site *i* at time *t*, where *x*_*i*_(*t*) = 1 [*x*_*i*_(*t*) = 0] indicates that site *i* is completely full [completely empty] at time *t*. Thus, the vector 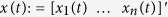 describes the density profile along the chain at time *t*. A parameter *λ*_*i*_ > 0, 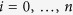, controls the transition rate from site *i* to site *i* + 1, where *λ*_0_ [*λ*_*n*_] is the initiation [exit] rate (see Fig. 1). The output rate at time *t* is *R(t*) = *λ*_*n*_*x*_*n*_(*t*). In the context of translation, the “material” are the moving ribosomes, and each site represents a group of codons, i.e. the mRNA is coarse-grained into *n* consecutive sites of codons. Thus, *R(t*), the output flow of ribosomes at time *t*, is also the *protein production rate* at time *t*. It is known^31^ that the RFM admits a unique *steady*-*state* production rate denoted by *R* = *R(λ*), where 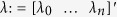.

Both TASEP and the RFM are phenomenological models of unidirectional transportation with excluded flow. The RFM approximates the equations describing the stochastic evolution of the site occupancy probabilities in TASEP by ignoring high-order correlations. This provides a good approximation except perhaps at specific parameter values where TASEP undergoes a sharp change (phase transition). The standard TASEP and the RFM also ignore the fact that the moving ribosome actually covers several codons. RFM with extended objects is an important research direction, but not pursued here. However, we show in the Appendix that the RFM, based on coarse graining of the mRNA molecule, provides predictions that agree well with the extended objects TASEP model.

It is important to note that mRNA translation has also been modeled and studied using more detailed stochastic models that include additional features such as both cognate tRNA-capture rates and translocation rates^32^, competition for near-cognate and non-cognate tRNAs^33^, and more. Typically, models with this level of detail can only be studied via simulations. An important advantage of the RFM is that it is amenable to rigorous analysis. This allows deriving general results that hold for any set of feasible parameters values. In order to confirm the validity of the results derived below using the RFM, we compare the results with those obtained using simulations of TASEP. We also include below a detailed example with parameter values derived from the *S. cerevisiae* gene *YBL025W* that encodes the protein *RRN10*.

Here we apply the RFM to analyze how to maximally down-regulate mRNA translation. To do this, we formulate the following general optimization problem. Given an mRNA molecule with *n* sites, and a convex and compact region of feasible transition rates Ω^*n*+1^, find a vector 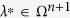 such that 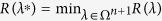. In other words, the problem is how to select transition rates, within a feasible region, such that the production rate is minimized (see Fig. 2). To the best of our knowledge, this is the first time that such a problem is analyzed in a dynamical model of mRNA translation.

As a concrete example, consider an RFM with dimension *n* and rates 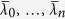. Given a “total reduction budget” 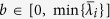, define the feasible set 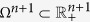 by


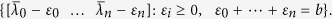


In other words, the feasible set is the set of all the rates obtained by reducing the rates of the given mRNA molecule by the total reduction equal to *b*. The question is how to distribute the total reduction budget over the rates so as to obtain the minimal possible protein production rate. We prove that:

- If some rate 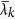 is a “bottleneck” rate, in a sense that will be made precise below, then an optimal reduction in protein production rate is obtained by using all the reduction budget *b* to further decrease 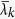;
- If all the given rates are equal, i.e. 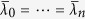, then the transition rate at the middle of the mRNA molecule is the bottleneck rate, and thus an optimal reduction in protein production rate is obtained by using all the reduction budget to reduce this transition rate.

Thus, in these cases there exists a single site such that mutating it yields the maximal inhibition of translation. Our results allow to determine where this site is located.

The remainder of this paper is organized as follows. We first briefly review some known results on the RFM that are needed for our purposes. The following section poses the problem of down-regulating the steady-state protein production rate in the RFM in an optimal manner, and then describes our main results. Analysis of the RFM is non-trivial, as this is a nonlinear dynamical model. In particular, the mapping from *λ* to *R(λ*) is nonlinear and does not admit a closed-form expression. Nevertheless, by combining tools from convex optimization and eigenvalue sensitivity theory, we show that this optimization problem is tractable in some cases, and rigorously prove several results that have interesting biological implications. The final section summarizes and describes several directions for further research. To increase the readability of this paper, all the proofs are placed in Section A in the Appendix. Section B in the Appendix includes a comparison between the RFM and extended objects TASEP predictions showing that the correlation between these two models is very high.

## Ribosome Flow Model

The dynamics of the RFM with *n* sites is given by *n* nonlinear first-order ordinary differential equations:


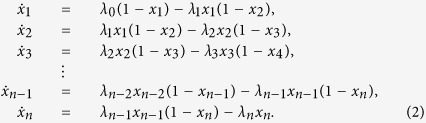


If we define 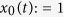 and 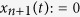 then (2) can be written more succinctly as


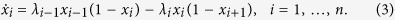


Eq. (3) can be explained as follows. The change of occupancy at site *i* at time *t* is equal to the flow of material from site *i* − 1 to site *i* minus the flow of material from site *i* to site *i* + 1 at time *t*. The latter is *λ*_*i*_*x*_*i*_(*t*) (1 − *x*_*i*+1_(*t*)). This flow is proportional to *x*_*i*_(*t*), i.e. it increases with the density at site *i*, and to (1 − *x*_*i*+1_(*t*)), i.e. it decreases as site *i* + 1 becomes fuller. This corresponds to a “soft” version of a simple exclusion principle. Note that the maximal possible flow from site *i* to site *i* + 1 is the transition rate *λ*_*i*_.

Let *x(t, a*) denote the solution of (2) at time *t* ≥ 0 for the initial condition *x*(0) = *a*. Since the state-variables correspond to normalized density levels, with *x*_*i*_(*t*) = 0 [*x*_*i*_(*t*) = 1] representing that site *i* is completely empty [full] at time *t*, we always assume that *a* belongs to the closed *n*-dimensional unit cube: 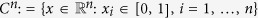. Let int(*C*^*n*^) [∂*C*^*n*^] denote the interior [boundary] of *C*^*n*^. It is straightforward to verify that ∂*C*^*n*^ is repelling, i.e. if 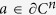 then 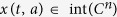 for all *t* > 0, so *C*^*n*^ and also int(*C*^*n*^) are invariant sets for the dynamics.

An important property of the RFM is the symmetry between the “particles” (i.e. ribosomes) moving from left to right and “holes” (i.e. “lack” of ribosomes) moving from right to left (in the TASEP literature, this property is sometimes referred to as the “particle-hole” symmetry). Indeed, let *q_j_(t*): = 1− *x*_*n* + 1− j_(*t*), 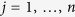. Then


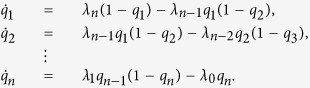


This is another RFM, but now with rates 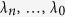.

The RFM has been used to model and analyze the flow of ribosomes along the mRNA molecule during the process of mRNA translation. The (soft) simple exclusion principle corresponds to the fact that ribosomes have volume and cannot overtake one another.

It has been shown in ref. 29 that the correlation between the production rate based on modeling using RFM and using TASEP over all *S. cerevisiae* endogenous genes is 0.96. In addition, it has also been shown there that the RFM agrees well with biological measurements of ribosome densities. Furthermore, it has also been shown that the RFM predictions correlate well (correlations up to 0.6) with protein levels in various organisms (e.g. *E. coli, S. pombe, S. cerevisiae*). Given the high levels of bias and noise in measurements related to gene expression and the inherent stochasticity of intracellular biological processes (see e.g. refs 34,35), these correlation values demonstrate the relevance of the RFM in this context.

### Steady-State Spectral Representation

Ref. 31 has shown that the RFM is a *tridiagonal cooperative dynamical system*^36^, and that (2) admits a *unique* steady-state point 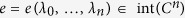 that is globally asymptotically stable, that is, 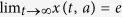 for all 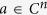 (see also ref. 37). This means that the ribosomal density profile always converges to a steady-state profile that depends on the rates, but not on the initial condition. In particular, the production rate *R(t*) = *λ*_*n*_*x*_*n*_(*t*) converges to a steady-state value 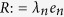.

At steady-state (i.e., for *x* = *e*), the left-hand side of all the equations in (2) is zero, so


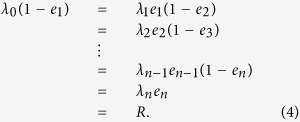


This yields


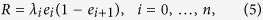


where 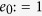 and 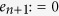. Ref. 38 used these expressions to provide a *spectral representation* of the mapping from the set of rates *λ* to the steady-state production rate *R*. Let 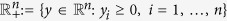 and 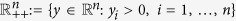.

**Theorem 1** [Ref. 38] *Given an RFM with rates* 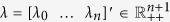, *let R* = *R(λ) denote its steady-state production rate. Define an* (*n* + 2) × (*n* + 2) *Jacobi matrix A* = *A(λ) by*


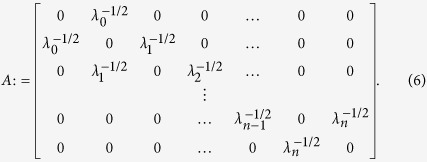


*Then*:

1. *The eigenvalues of A are real and distinct, and if we order them as* 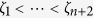 *then* 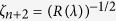.
2. *Let* 
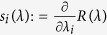
, *i.e. the sensitivity of R with respect to* (*w.r.t*.) *the rate λ*_*i*_. *Let* 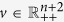 *denote an eigenvector of A corresponding to the eigenvalue ζ*_*n*+2_. *Then*

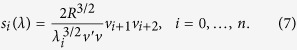


This means that the steady-state production rate, and its sensitivity with respect to the transition rates, can be computed efficiently using numerical algorithms for computing the eigenvalues and eigenvectors of tridiagonal matrices. Theorem 1 also implies that


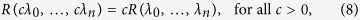


i.e. *R(λ*) is homogeneous of degree one.

Another important implication of Theorem 1 is that *R* is a *strictly concave* function of the transition rates 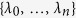 over 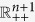
38. Also, it implies that 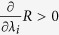 for all *i*, that is, an increase in any of the rates yields an increase in the steady-state production rate.

For more on the analysis of the RFM, and also networks of interconnected RFMs, using tools from systems and control theory, see e.g. refs 38, 39, 40, 41, 42, 43, 44.

## Main Results

We begin by posing a general minimization problem for the steady-state production rate in the RFM (see Problem 1 below). Using the concavity of the mapping from the rates *λ* to the steady-state production rate *R* implies that any solution to this problem must be an extremal point of the feasible set (see Proposition 1 below). We then show how various interesting biological problems can be cast as special cases of this general problem.

**Problem 1** *Given a convex and compact feasible set of transition rates* 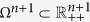, *find* 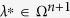 *such that* 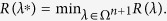


From the biological point of view, the feasible set of transition rates Ω^*n*+1^ depends on all the biophysical constraints on the transition rates along the coding sequence. For example, the maximal/minimal decoding rate of a codon (e.g. via its adaptation to the tRNA pool)^45^, the maximal possible effect of mRNA folding (after codon substitution) on each codon^46^, the maximal possible effect (after amino acid substitution) of the interaction of the ribosome with amino acids of the nascent peptide^47^, and the maximal possible elongation slow down due to interaction with microRNAs^9^,^10^.

Below we explain how to pose various interesting biological problems in the framework of Problem 1. Examples include finding the minimal number of mutations that down regulate translation of a gene/mRNA under a certain “total reduction budget”. This is practically important when we use costly (in terms of time and money) gene editing approaches. Another related question is how to down regulate translation of a gene/mRNA with a maximal number of mutations. This is important when attenuating viral replication rate for generating a safe live attenuated vaccine. A large number of mutations reduces the probability of reverting. One may also define the feasible set in Problem 1 in such a way that some rates cannot be changed. This is relevant for example when some codons along the mRNA cannot be modified. Indeed, various positions along the mRNA affect regulatory mechanisms that we may not want to alter (e.g. co-translational folding and splicing).

It is well-known (see, e.g. ref. 48, Thm. 7.42) that if 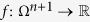 is a continuous and strictly convex function defined over a convex and compact set Ω^*n*+1^ then all the maximizers of *f* over Ω^*n*+1^ are extreme points of Ω^*n*+1^ (for more on the problem of maximizing a convex function, or equivalently, minimizing a concave function, see e.g. ref. 49). Combining this with the fact that *R* is a strictly concave function of the transition rates over 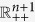 implies the following.

**Proposition 1** *Every solution of Problem 1 is an extreme point of* Ω^*n*+1^.

In particular, if the set of extreme points of Ω^*n*+1^ is finite then one can always solve Problem 1 by simply calculating *R(λ*) for all *λ* that are extreme points of Ω^*n*+1^, and then finding the minimum of these values. In particular, if Ω^*n*+1^ is a convex polytope then the extreme points are just the vertices of Ω^*n*+1^. Thus, when the biophysical constraints lead to a feasible set of rates that is a convex polytope then it is computationally straightforward to determine how to modify the rates so as to obtain the largest decrease in production rate under reasonable biophysical constraints.

In the remainder of this section, we consider three special cases of Problem 1 for which it is also possible to obtain analytic results.

**Problem 2** *Given an RFM with n sites, rates* 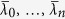, *and a total reduction budget* 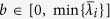, *let* 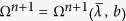 *be the set*


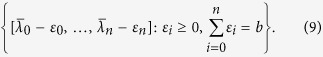


*Find* 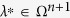 *such that* 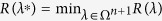.

In other words, Ω^*n*+1^ is the set of all the rates that can be obtained by applying a total reduction *b* to the given rates 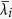. From a mathematical point of view, *b* provides a bound on the total possible rate reduction. It also couples the reduction in different rates, as a larger reduction in one rate must be compensated by smaller reductions in the other rates so that the total reduction will not exceed *b*. From a synthetic biology point of view, *b* can be used to capture the idea of maximally inhibiting the production rate while minimizing the side-effects of this down regulation. For example, a very small value of *b* forces a solution with small modifications in all the rates. This is expected of course to minimize the effect of the mutations on the fitness of the cell/organism. Since co-translational folding^50^,^51^,^52^ is related to the ribosome transition rates along the mRNA, smaller changes in the rates are expected to have a smaller effect on protein folding (and thus on the functionality of the protein and the overall organismal fitness). Smaller changes in the transition rates are also related to a “simpler” biological solution in the sense of fewer mutations, less miRNAs, etc.

The next example demonstrates Problem 2.

**Example 1** Consider an RFM with length *n* = 4 and transition rates


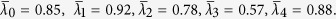


The steady-state production rate is 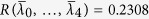 (all numbers are to four digit accuracy). Suppose that the total reduction budget is *b* = 0.1. Then, for example, the vector


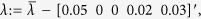


belongs to Ω^5^, and *R(λ*) = 0.2260. An optimal solution for Problem 2 is


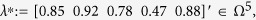


with R(λ*) = 0.2140. Note that this corresponds to reducing *b* from the rate 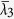, which is the minimum of all the rates 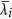, leaving all the other rates unchanged. 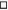


Let 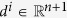 denote the (*i* + 1)’th column of the (*n* + 1) × (*n* + 1) identity matrix. The set 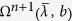 is a convex polytope with vertices:


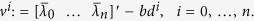


If there exists an index *i* such that 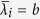 then it is clear that an optimal solution is to reduce 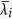 to 0, as then the steady-state production rate will be zero. So we always assume that *b* takes values in the set 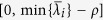, for some *ρ* > 0. This means that Problem 2 is a special case of Problem 1, as 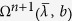 is a convex polytope contained in 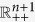.

By Prop. 1, every solution of Problem 2 is contained in the set 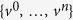. In other words, every minimizer corresponds to reducing *all* the available budget *b* from a single rate. This immediately yields a simple and efficient algorithm for solving Problem 2: use the spectral representation of *R* to compute *R(v*^*i*^), 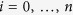, and then find the minimum of all these values. Since the matrix *A* in (6) is symmetric and tridiagonal, calculating *R(v*^*i*^) can be done efficiently even for large values of *n*. We wrote a simple (and unoptimized) MATLAB script for solving Problem 2, and ran it on a MAC laptop with a 2.6 GHz Intel core *i*7 processor. For an RFM with *n* = 500 (a typical coding region includes a few hundred codons^53^), rates 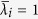, 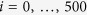, and *b* = 0.1, the optimal solution is found in 3.14 seconds.

Example 1 may suggest that reducing the slowest transition rate by *b* always yields an optimal solution, but in general this is not true (see Example 3 below).

One may also consider a different feasible set in Problem 2, namely,


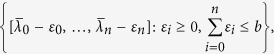


i.e. here the total reduction is *up to b*. However, by Theorem 1 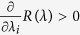 for all *i*, and thus an optimal solution for this problem is guaranteed to agree with an optimal solution of Problem 2.

The next example demonstrates the effect of increasing the total reduction rate *b* on the optimal solution of Problem 2. As noted above, we also compare the results derived for the RFM to TASEP simulations. The simulations used throughout this paper use a parallel update mode. At each time tick *t*_*k*_, the sites along the lattice are scanned from site *N* backwards to site 1. If it is time to hop, and the consecutive site is empty then the particle advances to the consecutive site. If the consecutive site is occupied the next hopping time, *t*_*k*_ + *ε*_*k*_, is generated randomly. For site *i, ε*_*k*_ is exponentially distributed with parameter (1/*μ*_*i*+1_) (see (1)). The occupancy at each site is averaged throughout the simulation, with the first 10^7^ cycles discarded in order to obtain the steady-state value. Let 
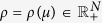
 denote the averaged occupancy and 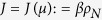 denote the averaged current (or output rate).

**Figure 3:**
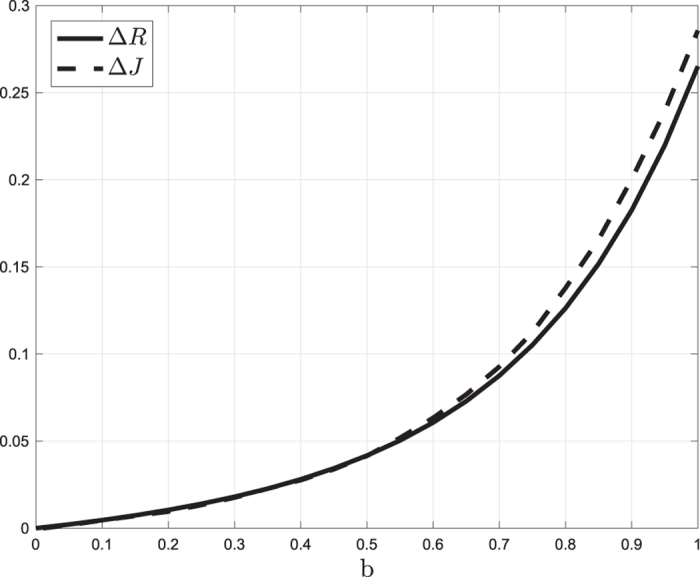
Δ*R* and Δ*J* as a function of *b* in Example 2.

**Example 2** Consider an RFM with dimension *n* = 10, and rates 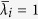, 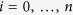. Here 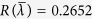. We calculated the optimal solution λ* for different values of *b*, and also the value 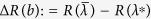, that is, the optimal reduction in the protein production rate that can be obtained for various values of *b*. We also simulated TASEP with *N* = 10, 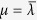, and also with the optimal solution 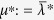, for different values of *b*, and computed the value 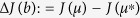. Figure 3 depicts Δ*R* and Δ*J* as a function of *b*. It may be seen that Δ*R* and Δ*J* increase quickly with *b* (specifically, the relation is superlinear), and that Δ*R* provides a good approximation of Δ*J*. 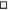


### Optimal reduction and sensitivities

It is also possible to derive theoretical results on the structure of an optimal solution λ* in Problem 2 using the sensitivities 
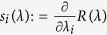
 (that can be computed efficiently using (7)). Recall that the proofs of all the analysis results are placed in Section A in the Appendix.

**Proposition 2** *Consider Problem 2. If there exist i*, 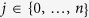 *such that*


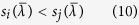


then any optimal solution λ* *satisfies* 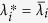.

In other words, if the sensitivity of the steady-state production rate to rate *λ*_*i*_ at 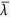 is lower than some other sensitivity then an optimal solution will *not* include a reduction in 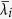. Indeed, it is better to distribute the reduction budget over some other, more sensitive, rates.

**Remark 1** *Note that since R is a strictly concave function of the rates*,


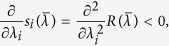


*for any* 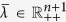 *and any* 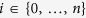. *In other words, a decrease in* 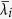 *increases the sensitivity w.r.t. this rate*.

Proposition 2 leads to the following definition.

**Definition 1** Given an RFM with rates 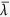, a transition rate 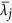 is called a bottleneck rate if 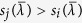, for all 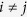.

In other words, a bottleneck rate is one with a maximal sensitivity.

Combining this with Proposition 2 immediately yields the following result.

**Corollary 1** *Given an RFM with rates* 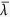, *suppose that* 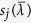 *is a bottleneck rate. Then the unique optimal solution to Problem 2 is obtained by reducing* 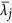 *by b*.

An important observation is that the slowest rate along the mRNA molecule and the bottleneck rate may be different. The next example demonstrates this.

**Example 3** Consider an RFM with dimension *n* = 4, and rates 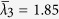, 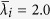, *i* = 0, 1, 2, 4. In this case, 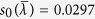, 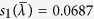, 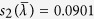, 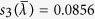, and 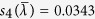. Thus, although the minimal rate is 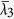, the bottleneck rate is 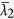. In particular, the optimal solution will be to reduce 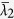 by *b*, and not 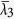, even though 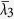 is the minimal rate. 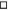


However, note that Remark 1 implies that if some rate *λ*_*i*_ is decreased enough then it will eventually becomes a bottleneck rate.

Proposition 2 can be used to derive analytic results in cases where we can obtain explicit information on the sensitivities at a point 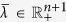. The next two results demonstrate this.

**Proposition 3** *Consider an RFM with dimension n and with equal rates, i.e*. 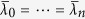. *If n is even then the unique optimal solution to Problem 2 is*: 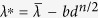. *If n is odd then there are two optimal solutions*: 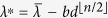 *and* 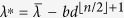.

In other words, in the case where all the rates are equal, the bottleneck is at the center of the chain. These results are closely related to the fact that in a dynamic model for phosphorelay^54^, that is very similar to the RFM, the middle layer in the model is the most sensitive to changes in the input. This also agrees with the so called “edge-effect” in the HTASEP^55^,^56^,^57^, i.e. the fact that the steady-state output rate is less sensitive to the rates that are close to the edges of the chain. For more on the sensitivity of TASEP to manipulations in the initiation, hopping, and exit rates, see refs 57, 58, 59, 60.

Another case where analytic results can be derived is when the rates in the RFM lead to equal steady-state occupancies along the mRNA molecule. This happens when 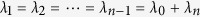 (see (4)).

**Proposition 4** *Consider an RFM with dimension n and rates* 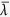 *such that* 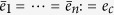, *i.e. all the steady-state occupancies are equal, and e*_*c*_ *denotes their common value*.

1. *If e*_*c*_ < 1/2 *then the unique optimal solution to Problem 2 is*
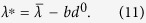
2. *If e*_*c*_ > 1/2 *then the unique optimal solution to Problem 2 is*
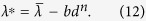
3. *If e*_*c*_ = 1/2 *then* (11) *and* (12) *are the optimal solutions*.

In other words, if the equal occupancy is relatively low [high] then maximal inhibition of the production rate is obtained by reducing the total reduction rate from the initiation [exit] rate, leaving all the other rates unchanged.

**Example 4** Consider Problem 2 for an RFM with *n* = 5, rates 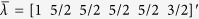, and *b* = 1/2. Note that in this case 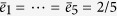. A calculation yields 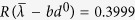, 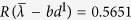, 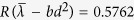, 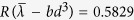, 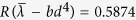, and 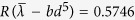, so the optimal solution is 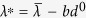. Since *e*_*c*_ < 1/2, this agrees with Proposition 4. We also simulated TASEP with *N* = 5, 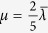 (this scaling is used to make *μ*_*i*_ ≤ 1 for all *i*), and a reduction budget 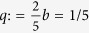. The TASEP simulation yields *J(μ* − *qd*^0^) = 0.1595, *J(μ* − *qd*^1^) = 0.2302, *J(μ* − *qd*^2^) = 0.2324, *J(μ* − *qd*^3^) = 0.2337, *J(μ* − *qd*^4^) = 0.2344, and *J(μ* − *qd*^5^) = 0.2221, so also for TASEP the optimal solution is μ* = *μ* − *qd*^0^. 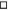


In some cases it may be more natural to define the transition rate reduction in relative rather than absolute terms. This is captured by the following optimization problem.

**Problem 3** *Given an RFM with n sites, rates* 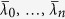, *and a total reduction budget* 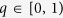, *let* 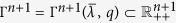 *be the set*


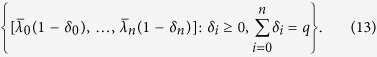


*Find* 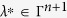 *such that* 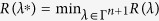.

For 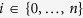, let 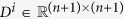 denote the (*n* + 1) × (*n* + 1) identity matrix, but with entry (*i* + 1, *i* + 1) changed to 1 − *q*. The set 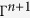 is a convex polytope with vertices 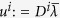, 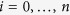. Thus, Problem 3 is also a special case of Problem 1, and so the minimizer λ* satisfies 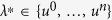.

In practice, each codon (or coding region) admits a minimal and a maximal possible decoding rate. There are also minimal and maximal values for the initiation rate. These bounds are determined by the biophysical properties of the transcript and the intracellular environment. To model this, we can modify the optimization problems described above to include a bound 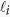 on the maximal allowed reduction of rate *i*, for 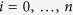. The next problem demonstrates such a modification for Problem 2.

**Problem 4** *Consider an RFM with n sites and rates* 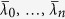. *Given a total reduction budget* 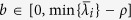, *for some ρ* > 0, *and also bounds* 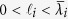, 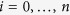, *with* 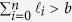, *let* Ω^*n*+1^ *be as defined in Problem 2, and let*


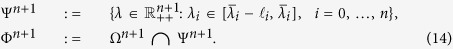


*Find* 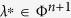 *such that* 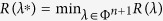.

In other words, the feasible set 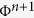 in Problem 4 is the intersection of the set Ω^*n*+1^ (defined in Problem 2), and the closed (*n* + 1)-dimensional cube 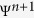 that models constraints on the maximal possible reduction of each rate.

Since 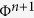 is compact and convex (being the intersection of two compact and convex sets), Problem 4 admits a solution that is an extreme point of 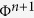. In general, not all the rates can be reduced by *b*, and thus an optimal solution may include a reduction of *several* rates.

**Figure 4:**
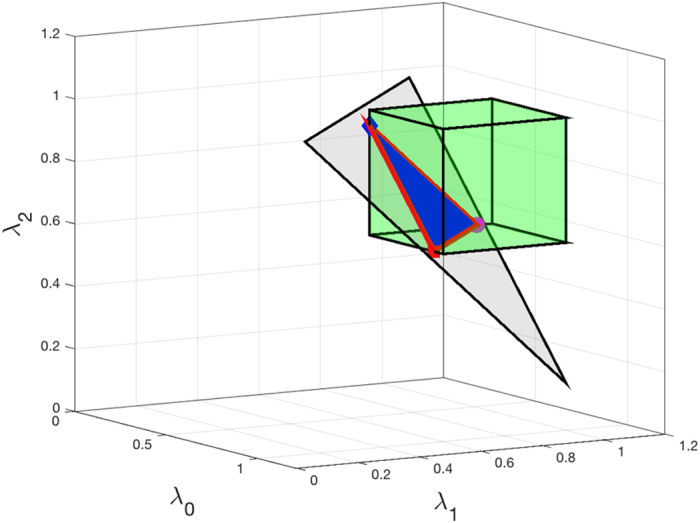
The sets Ω^3^ (gray polytope), Ψ^3^ (green cube), and Φ^3^ (blue polytope) in Example 5.

**Example 5** Consider Problem 4 for an RFM with dimension *n* = 2, rates 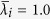, *i* = 0, 1, 2, and parameters *b* = 0.85, and 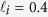, *i* = 0, 1, 2. In other words, the total possible reduction is 0.85, but any rate can be reduced by no more than 0.4. Figure 4 depicts the feasible set 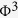 (blue polytope) that is the intersection of the set Ω^3^ (gray polytope) and the set 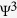 (green cube). Shown also are the three extreme points of 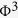:


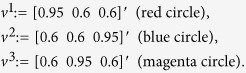


A calculation yields *R(v*^1^) = *R(v*^2^) = 0.2538, whereas *R(v*^3^) = 0.2764. It follows that λ* = *v*^1^ and λ* = *v*^2^ are optimal solutions. Note that these solutions correspond to reducing several rates along the mRNA molecule. Note also that 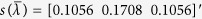, so both optimal solutions correspond to a maximal possible reduction in a most sensitive rate, and a maximal possible reduction in another most sensitive rate. 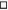


In some cases, there may be positions along the coding region that we cannot modify due to their potential effect on various intracellular processes. An important advantage of Problem 4 is that it allows capturing this by simply setting some of the 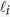
s to zero. If the problem with the additional constraints can be described as a special case of the general Problem 4 then we can efficiently determine the optimal solution.

On the other hand, in down regulation of a viral gene it may be desirable to distribute the synonymous codon modifications over many mRNA sites in order to reduce the chance of spontaneous mutations yielding the original wild type. This is captured by Problem 4 when we set the 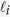
s to small non-zero values, as then an optimal solution will include a transition rate reduction in many sites.

### A biological example

To demonstrate how the results above can be used to analyze translation and provide guidelines for re-engineering the mRNA, we consider the *S. cerevisiae* gene *YBL025W* that encodes the protein *RRN10*, which is related to regulation of RNA polymerase I. This gene has 145 codons (excluding the stop codon). Similarly to the approach used in ref. 29, we divided this mRNA into 6 consecutive pieces: the first piece includes the first 24 codons (that are also related to later stages of initiation^52^). The other pieces include 25 non-overlapping codons each, except for the last one that includes 21 codons. This partitioning was found to optimize the correlation between the RFM prediction and biological data measurements.

To model this using an RFM with *n* = 5 sites, we first estimated the elongation rates 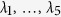 using ribo-seq data for the codon decoding rates^45^, normalized so that the median elongation rate of all *S. cerevisiae* mRNAs becomes 6.4 codons per second^61^. The site rate is (site time)^−1^, where site time is the sum over the decoding times of all the codons in this site. These rates thus depend on various factors including availability of tRNA molecules, amino acids, Aminoacyl tRNA synthetase activity and concentration, and local mRNA folding^1^,^45^,^52^. Note that if we replace a codon in a site of mRNA by a synonymous slower codon then the decoding time increases and thus the rate associated with this site decreases.

The initiation rate (that corresponds to the first piece) was estimated based on the ribosome density per mRNA levels, as this value is expected to be approximately proportional to the initiation rate when initiation is rate limiting^29^,^62^. Again we applied a normalization that brings the median initiation rate of all *S. cerevisiae* mRNAs to be 0.8^63^. Adding the initiation time (1/0.4482) to the site time of the first piece yields an RFM with *n* = 5 and parameters:


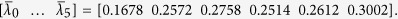


A calculation yields that the steady-state production rate in this RFM is *R* = 0.0732. We also simulated TASEP with *N* = 5 and 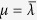, and for these values the simulation yields the steady-state current *J* = 0.0776.

In order to analyze the solution of Problem 2 for this RFM we calculated the sensitivities using (7). This yields: 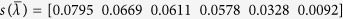, so 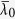 is a bottleneck rate. This means that the solution for Problem 2 is to reduce all the reduction budget *b* from 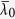. In biological terms, this suggests that maximal inhibition of production should be based on replacing some (or all) of the first 24 codons with slower synonymous codons. For comparison with the optimization scenarios described below, consider the total budget *b* = 0.0089. The solution for Problem 2 is then to reduce *λ*_0_ by *b*, and this yields


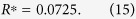


Reducing *λ*_0_ by *b* in the model is possible by substituting codons in the first site with their slowest synonymous mutation (for example, the third codon AGA should be replaced by the synonymous codon CGG, increasing the codon decoding time from 0.1128 seconds to 0.2246 seconds). A TASEP simulation with *α* reduced by *b* yields


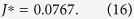


Now suppose that we are not interested in modifying these codons because in this region there are various regulatory signals that we may not want to change (see, for example, ref. 52). To maximize inhibition of production rate under this constraint, we apply Problem 4, with 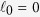, and 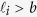 for all 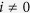. Now the optimal solution is to reduce *b* from 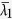. Note that 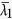 has the second largest sensitivity. This yields R* = 0.0726, and is, as expected, higher than the value in (15). Again, the biological data shows that such a reduction can be done by synonymously replacing codons 34 (GCT with GCA), 35 (GTT with GTA), 36 (CCT with CCC), 38 (CCG with CCC), 39 (TTC with TTT), and 49 (GTG with GTA). A TASEP simulation with *γ*_1_ reduced by *b* yields *J* = 0.0769, which is indeed higher than the value in (16).

Finally, to demonstrate mutations in multiple sites, we used the data to find a scenario where a set of mutations yields the same total decrease in the rates. This can be done by synonymously replacing codons 21 (GTG with GTA), 29 (GAA with GAG), 58 (TTC with TTT), 82 (AAG with AAA), 110 (CTA with CTG), and 141 (GCG with GCA), leading to


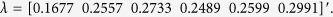


Note that all the rates are reduced and that the total reduction is *b*. This yields *R* = 0.0727, which is again higher than the value in (15). A TASEP simulation with *μ* = *λ* yields *J* = 0.0771, which is again higher than the value in (16).

## Discussion

There are several approaches for effectively down-regulating translation. Global down-regulation can be achieved by controlling basic translation factors or by using drugs that induce ribosome stalling^64^,^65^,^66^.

Here we consider down regulation of specific genes via targeting specific codons/regions in these genes. This leads to the problem of finding the codon regions that have the most effect on the steady-state production rate. We pose this problem of optimal down regulation of mRNA translation as a general optimization problem (Problem 1 above) for a mathematical model for ribosome flow, the RFM. All possible modifications of the rates define a feasible set of rates, and, under certain conditions, we give a simple algorithm for finding the optimal solution, that is, the rates that lead to a maximal decrease in the protein production rate. For some specific cases, we also derive theoretical results on the optimal solution. Our general formulation thus provides guidelines on how to pose the optimal down regulation problem properly.

Our results show that the solution must focus on the positions along the mRNA molecule where the transition rate has the strongest effect on the protein production rate. However, this position is not necessarily the one with the minimal rate (though in many cases there are correlations between the two definitions). Many previous studies in the field emphasized the importance of the translation bottleneck^22^,^59^,^67^, however, this is always defined as the minimal rate. We believe that the sensitivity of the coding region sites should be further studied in order to understand better the evolution of transcripts and their design.

In addition, we demonstrated using TASEP simulations that the analytical results obtained for the RFM may be used to obtain an optimal solution in the nonhomogeneous TASEP as well. This is important since TASEP and its many variants are employed to analyze many biological (and other) phenomena, and the analytical results obtained in this paper may be useful in these cases as well.

The optimization problems posed here are flexible enough to capture various scenarios. For example, in some cases it may be desirable to introduce a *minimal* number of changes in the transcript to obtain the desired decrease in the translation rate. Indeed, generating mutations and using suitable RNAi molecules is costly in time and money. Also, any change in the translation rates can affect various important phenomena such as co-translational folding^50^,^51^,^52^, as well as other properties that are encoded in the coding region^52^,^68^,^69^. In other cases, such as generating a down-regulated virus strain, it may be desirable to introduce as many mutations as possible.

There are various approaches for synthesizing molecules that block mRNA translation (see e.g. http://www.gene-tools.com/choosing_the_optimal_target). In practice, when determining an optimal position to target (e.g. with RNAi molecules) one must take into account additional biophysical aspects. For example, the GC content at the different regions along the mRNA, the folding of the mRNA, the potential binding affinity of the RNAi and the mRNA, potential un-desired binding of the RNAi to additional mRNAs or regions within the mRNA, etc. Nevertheless, we feel that our results can be integrated to improve the design of such tools.

In addition, it is entirely possible that in the future this downregulation problem will be studied using other and perhaps more detailed models of translation. For example, in practice, there are many mRNA molecules in the cell and they all compete for the finite pool of free ribosomes. In particular, if more ribosomes are stuck in a traffic jam on a certain mRNA molecule then the pool of free ribosomes is depleted yielding a reduction in the production rates in other mRNA molecules. The RFM is a model for ribosome flow along a single isolated mRNA molecule. This is a reasonable model when the expression levels (e.g. the mRNA levels and the total number of ribosomes on the mRNA molecules related to the gene) are relatively low, so that changes in the translation dynamics on one mRNA have a negligible effect on the pool of ribosomes and thus on the other mRNAs. A model for a network of RFMs, interconnected via a dynamic pool of free ribosomes, has been studied in ref. 44. It may be of interest to study the problem of down regulation of a specific mRNA molecule within this framework. In this case, one can also down regulate the mRNA indirectly by affecting the ribosomal pool. However, the analytic tools used here do not directly apply, as the convexity results for a single chain do not necessarily carry over to the case of a network of RFMs.

The results here suggest several biological experiments for studying the problem of optimal down regulation and, in particular, validating the theoretical predictions derived using the RFM. Libraries encoding the same protein using mRNAs with different codons (but similar mRNA levels and translation initiation rates) can be generated as was done in ref. 17. For each variant the protein levels, that are expected to monotonically increase with the production rate^29^, can be measured either via a reporter protein^17^ or directly^70^. The codon decoding rates can be estimated based on ribo-seq experiments^17^,^45^. Such an experimental testbed can be used to validate the results reported in this study.

## Additional Information

**How to cite this article**: Zarai, Y. *et al*. Optimal Down Regulation of mRNA Translation. *Sci. Rep.* **7**, 41243; doi: 10.1038/srep41243 (2017).

**Publisher's note:** Springer Nature remains neutral with regard to jurisdictional claims in published maps and institutional affiliations.

## Supplementary Material

Supplementary Information

## Figures and Tables

**Figure 1:**
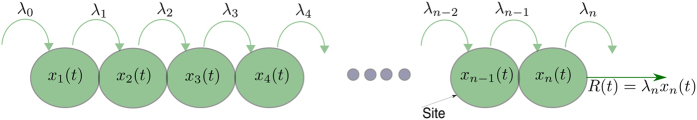
The RFM models unidirectional flow along a chain of *n* sites. The state variable 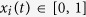 represents the density at site *i* at time *t*. The parameter *λ*_*i*_ > 0 controls the transition rate from site *i* to site *i* + 1, with *λ*_0_ > 0 [*λ*_*n*_ > 0] controlling the initiation [exit] rate. The output rate at time *t* is *R(t*) = *λ*_*n*_*x*_*n*_(*t*).

**Figure 2:**
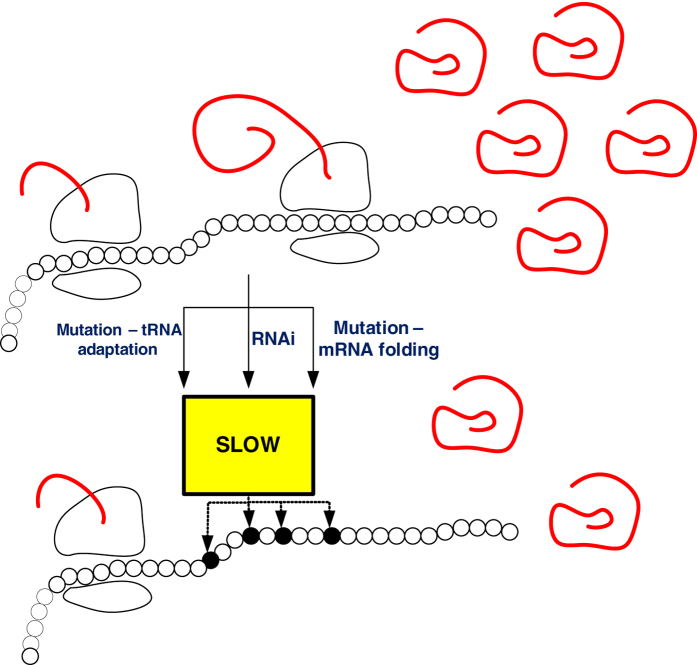
The problem we consider is how to efficiently select transition rates along the mRNA molecule, within a given set of possible rates, such that the protein production rate is minimized. In practice, translation rate modification can be done by introducing mutations into the gene or by designing a corresponding RNAi molecule.
