# Supplementary material for: Optimal Down Regulation of mRNA Translation
Source: Sci Rep. 2017 Jan 25;7:41243. doi: 10.1038/srep41243 (PMC5264618; doi:10.1038/srep41243)
Supplement: Supplementary Information [file srep41243-s1.pdf]

Appendix: Optimal Down Regulation of mRNA Translation

Yoram Zarai, Michael Margaliot and Tamir Tuller

A. Proofs

Proof of Proposition 2. Consider Problem 2, and suppose that (11) holds. We need to show that $\lambda_i^* = \bar{\lambda}_i$. Seeking a contradiction, assume that $\lambda_i^* < \bar{\lambda}_i$. By Prop. 1, $\lambda^* = \bar{\lambda} - bd^i$, so in particular $R(\bar{\lambda} - bd^i) \leq R(\bar{\lambda} - bd^j)$. Since R is a homogeneous function of the rates, we conclude that $R(c\bar{\lambda} - cbd^i) \leq R(c\bar{\lambda} - cbd^j)$ for any $c > 0$. Now taking $c > 0$ sufficiency small yields $\frac{\partial R(\bar{\lambda})}{\partial \lambda_i} \geq \frac{\partial R(\bar{\lambda})}{\partial \lambda_j}$. This contradicts (11). ■

Proof of Proposition 3. In the case where all the rates are equal there exists a closed-form expression for the sensitivities [1], namely,

$$s_i = \frac{\sin\left(\frac{i+1}{n+3}\pi\right)\sin\left(\frac{i+2}{n+3}\pi\right)}{2(n+3)\cos^3\left(\frac{\pi}{n+3}\right)}, \quad i = 0, \dots, n.$$

This means that

$$s_i = \frac{a - \cos\left(\frac{2i+3}{n+3}\pi\right)}{b}, \tag{1}$$

where $a, b > 0$ are constants that do not depend on i. If n is even then the cosine function in (1) admits a unique minimum at $i = n/2$, and combining this with Proposition 2 completes the proof. If n is odd then the cosine function in (1) admits two minima: at $\lfloor n/2 \rfloor$ and at $\lfloor n/2 \rfloor + 1$. Now arguing as in the proof of Proposition 2 and using the particle-hole symmetry of the RFM completes the proof. ■

Proof of Proposition 4. If $\bar{e}_1 = \dots = \bar{e}_n := e_c$, then (3) yields

$$\bar{\lambda}_i = \begin{cases} 1, & i = 0, \\ e_c^{-1}, & i = 1, \dots, n-1, \\ e_c^{-1} - 1, & i = n, \end{cases} \tag{2}$$

where we scaled $\bar{\lambda}_0$ to one w.l.o.g. In this case, the Perron eigenvector $v \in \mathbb{R}^{n+2}_{++}$ of the matrix $A(\bar{\lambda})$ is given by (see also [1]):

$$v_i = \begin{cases} 1, & i = 1, \\ \mu^{(i-1)/2} e_c^{-1/2}, & 2 \leq i \leq n+1, \\ \mu^{n/2}, & i = n+2, \end{cases} \tag{3}$$

where $\mu := e_c/(1 - e_c)$. We consider two cases.

The research of MM and TT is partially supported by a research grant from the Israeli Ministry of Science, Technology, and Space and the US-Israel Binational Science Foundation.

Y. Zarai is with the School of Electrical Engineering, Tel-Aviv University, Tel-Aviv 69978, Israel. E-mail: ravehalon@gmail.com
M. Margaliot is with the School of Electrical Engineering and the Sagol School of Neuroscience, Tel-Aviv University, Tel-Aviv 69978, Israel. E-mail: michaelm@eng.tau.ac.il
T. Tuller is with the Dept. of Biomedical Engineering and the Sagol School of Neuroscience, Tel-Aviv University, Tel-Aviv 69978, Israel. E-mail: tamirtul@post.tau.ac.il

If $e_c = 1/2$ then $v'v = 2(n+1)$ and applying Theorem (1) yields the sensitivities:

$$s_i = \begin{cases} \frac{1}{2(n+1)}, & i = 0, \\ \frac{1}{4(n+1)}, & 1 \le i \le n-1, \\ \frac{1}{2(n+1)}, & i = n. \end{cases} \tag{4}$$

Thus, $s_0 = s_n > s_j$, for all $j \notin \{0, n\}$, and arguing as in the proof of Proposition 2 and using the particle-hole symmetry implies that the two optimal solutions are $\bar{\lambda} - bd^0$ and $\bar{\lambda} - bd^n$.

If $e_c \neq 1/2$ then Theorem 1 yields

$$s_i = \begin{cases} \frac{1-2e_c}{1-\mu^{n+1}}, & i = 0, \\ \frac{e_c(1-2e_c)}{1-\mu^{n+1}}\mu^i, & 1 \le i \le n-1, \\ \frac{\mu^{n+1}(1-2e_c)}{1-\mu^{n+1}} & i = n. \end{cases} \tag{5}$$

When $e_c < 1/2$ [$e_c > 1/2$] (5) yields $s_0 > s_j$, for all $j \neq 0$ [$s_n > s_j$, for all $j \neq n$]. Combining this with Proposition 2 completes the proof. ■

B. TASEP and RFM Comparison

To show that the predictions obtained using the RFM, and specifically the results obtained in this paper, are biologically relevant, we compared the predictions of RFM and TASEP using real biological data as follows. We considered 100 *S. cerevisiae* genes with various mRNA levels (all genes were sorted according to their mRNA levels, and 100 genes were uniformly sampled from the list).

Similarly to the approach presented in the biological example section in the main text, to model the translation dynamics in these mRNAs using the RFM we divided the mRNAs related to these genes to non-overlapping pieces. Here we choose the piece size to be 10 codons, which is the size of the ribosome footprint (other than the first piece that includes 9 codons, and the last piece that includes between 5 and 15 codons). The estimation of the codons decoding times, elongation rates, and initiation rates were done as described in the biological example section in the main text.

To model the translation dynamics in these mRNAs using TASEP, we simulated *TASEP with extended objects* [2], where each site is a codon, and where the ribosome size was set to 10 codons.

Figure B.1 depicts the correlation between the steady-state production rates in the TASEP and the RFM over the 100 genes. The Pearson correlation coefficient, and the corresponding p-value are also given in the figure, indicating that the correlation between the two models is close to perfect. Figure B.2 depicts the correlation between the steady-state mean coverage density in the TASEP and the RFM over the same 100 genes. Again, in this case the correlation between the two models is close to perfect. These results validate the predictions obtained by the RFM.

References

[1] G. Poker, M. Margaliot, and T. Tuller, "Sensitivity of mRNA translation," *Sci. Rep.*, vol. 5, no. 12795, 2015.

[2] L. B. Shaw, R. K. P. Zia, and K. H. Lee, "Totally asymmetric exclusion process with extended objects: a model for protein synthesis," *Phys. Rev. E*, vol. 68, p. 021910, 2003.

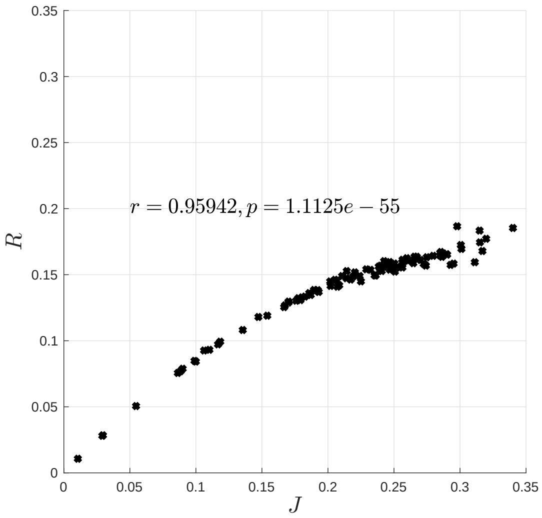

Fig. B.1. Steady-state production rate in the RFM (R) vs. extended objects TASEP (J) over the 100 genes. Also shown are the Pearson correlation coefficient r value and the p-value.

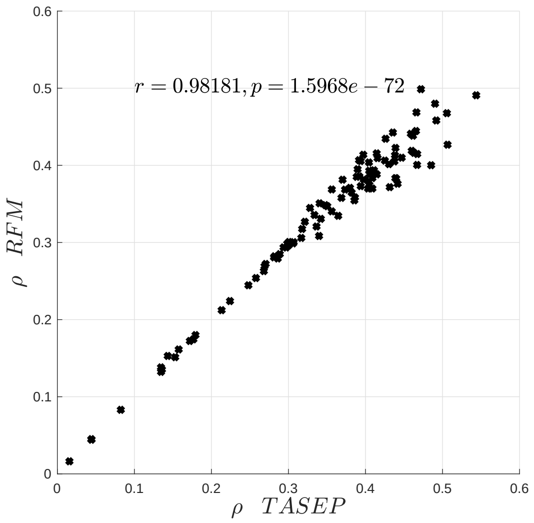

Fig. B.2. Steady-state mean coverage density in the RFM vs. extended objects TASEP over the 100 genes. Also shown are the Pearson correlation coefficient r value and the p-value.
